# Supplementary material for: Identification and Copy Number Variant Analysis of Enhancer Regions of Genes Causing Spinocerebellar Ataxia
Source: Int J Mol Sci. 2024 Oct 18;25(20):11205. doi: 10.3390/ijms252011205 (PMC11508295; doi:10.3390/ijms252011205)
Supplement: Supplementary file 1 [file ijms-25-11205-s001.zip › ijms-3223219-supplementary.pdf]

Supplementary Tables

Supplementary Table S1. Identified PutEs, R-PutEs and in vitro-active enhancers of *ATXN1*, *ATXN3*, *TBP* and *ITPR1*.

Gene	PutEs	Location (Hg19)	R-PutEs	In vitro-active enhancer
ATXN1	PutE1	chr6:16319229-16323196	Yes	Yes
	PutE2	chr6:16803696-16804514	Yes	Yes
ATXN3	PutE1	chr14:91975209-91975777	Yes	Yes
	PutE2	chr14:92505300-92508216	Yes	Yes
	PutE3	chr14:93069393-93070523	No	-
	PutE4	chr14:93214153-93215641	Yes	No
	PutE5	chr14:93259723-93261534	No	-
TBP	PutE1	chr6:170151018-170153022	Yes	Yes
	PutE2	chr6:170189867-170191217	Yes	Yes
ITPR1	PutE1	chr3:3839946-3843237	No	-
	PutE2	chr3:4344258-4346242	Yes	Yes
	PutE3	chr3:4508178-4509659	No	-
	PutE4	chr3:4761716-4763874	No	-
	PutE5	chr3:4791018-4794112	No	-
	PutE6	chr3:4908570-4911348	No	-
	PutE7	chr3:5018875-5022761	Yes	Yes

Supplementary Table S2. Location of the promoters and PutEs of *ATXN1*, *ATXN3*, *TBP* and *ITPR1* with respect to the TADs.

Gene	Region	Location (Hg19)	Associated TAD
ATXN1	Promoter	chr6:16759685-16763685	chr6:16200000-16760000 and chr6:16760000-17120000
	PutE1	chr6:16319229-16323196	chr6:16200000-16760000
	PutE2	chr6:16803696-16804514	chr6:16760000-17120000
ATXN3	Promoter	chr14:92570908-92574908	chr14:91980000-93260000
	PutE1	chr14:91975209-91975777	chr14:91980000-93260000
	PutE2	chr14:92505300-92508216	chr14:91980000-93260000
	PutE3	chr14:93069393-93070523	chr14:91980000-93260000
	PutE4	chr14:93214153-93215641	chr14:91980000-93260000
	PutE5	chr14:93259723-93261534	chr14:91980000-93260000
TBP	Promoter	chr6:170861456-170865456	chr6:169280000-170900000
	PutE1	chr6:170151018-170153022	chr6:169280000-170900000
	PutE2	chr6:170189867-170191217	chr6:169280000-170900000
ITPR1	Promoter	chr3:4533031-4537031	chr3:3220000-4680000
	PutE1	chr3:3839946-3843237	chr3:3220000-4680000
	PutE2	chr3:4344258-4346242	chr3:3220000-4680000
	PutE3	chr3:4508178-4509659	chr3:3220000-4680000
	PutE4	chr3:4761716-4763874	chr3:4680000-6840000

	PutE5	chr3:4791018-4794112	chr3:4680000-6840000
	PutE6	chr3:4908570-4911348	chr3:4680000-6840000
	PutE7	chr3:5018875-5022761	chr3:4680000-6840000

Supplementary Table S3. Primers used to amplify R-PutEs of *ATXN1*, *ATXN3*, *TBP* and *ITPR1*, a previously reported validated enhancer (positive control) [12] and a random 1479 bp DNA fragment (negative control) for cloning into the pGL4.23 Firefly luciferase reporter vector. Primer sequences are shown in bold and the restriction sites in orange.

**R-PutEs**	**Cloned region (Hg19)**	**Primers Sequence (5′ → 3′)**	
ATXN1-R-PutE1	chr6:16319330-16322021	Forward	AAAAGATATC**TCCCTGTTCCTAGGGAGGCTGAAG**
		Reverse	TTTTAAGCTT**CTGACCTCATGCTAAGCGGCACAC**
ATXN1-R-PutE2	chr6:16803384-16804756	Forward	AAAAGATATC**TAGTGGGCAGTCATTGAAGTAGGG**
		Reverse	TTTTAAGCTT**CCAGAAGAGGAAAGGGAGGGTATT**
ATXN3-R-PutE1	chr14:91975120-91975888	Forward	AAAAGCTAGC**GCCAACTTCTCTGCACCTGTTAACC**
		Reverse	TTTTAGATCT**TGAAGGGCATGTCCCTGCTTGTC**
ATXN3-R-PutE2	chr14:92505311-92507475	Forward	AAAACTCGAG**TGGGCGAAATGGGCGATTATACAC**
		Reverse	TTTTAAGCTT**ACTGGAGGCATTCCCAATGTTCTG**
ATXN3-R-PutE4	chr14:93214097-93215784	Forward	AAAAGCTAGC**TTCACCACATACCACAGGGCTAGTC**
		Reverse	TTTTAGATCT**CCACCCAGAAACACCAACTCCAAAG**
TBP-R-PutE1	chr6:170150661-170152979	Forward	AAAACTCGAG**GGTCCACGAAGTAAGTACGAAGAG**
		Reverse	TTTTGATATC**GGCAGAGGTAAAGTTTAGCCTTCC**

TBP-R-PutE2	chr6:170189723-170191350	Forward		AAAAAAGCTT**CTGCAGAGTGTGACAGTGACATTCC**
		Reverse		TTTTGATATC**TAATAAGCCTGTGCTGCTGGCTTCG**
ITPR1-R-PutE2	chr3:4344253-4346282	Forward		AAAAAAGCTT**AGGGCATAATGAGGCATGTTGGAC**
		Reverse		TTTTGATATC**AGAAACTCAGGCTCTGGGAAGTAG**
ITPR1-R-PutE7	chr3:5019011-5020902	A	Forward	AAAAGATATC**CCAAGGCGACCTGAAGGAAATAC**
			Reverse	TTTTAAGCTT**CGGCTTCATCACATGAGTCTCAC**
	chr3:5020684-5022750	B	Forward	AAAAGATATC**TCGCCCACGTTACCAACTTCTCCC**
			Reverse	TTTTAAGCTT**AGCAGCCTCTGCCGACCTTAACTC**
Negative control	chr18:941593-943071	Forward		AAAACTCGAG**TGTGAACCACGTGTCCCTCTTAG**
		Reverse		TTTTGATATC**CCCTGTCATTAAGCGATGCATAGC**
Positive control	chr5:89704487-89706712	Forward		AAAACTCGAG**TGGAGCCGCAACACTGGAATAAC**
		Reverse		TTTTGATATC**AGCCCATCCTTCTAGGTCTAACG**

Supplementary Table S4. Promoter viewpoints and 4C-templates used for each viewpoint of *ATXN1*, *ATXN3*, *TBP* and *ITPR1*.

**Gene**	**Promoter viewpoint (Hg19)**	**4C-template**
ATXN1	Chr6:16761086-16761915	*NlaIII/Csp6I*
ATXN3	Chr14:92572894-92573491	*NlaIII/DpnII*
TBP	Chr6:170863371-170863896	*NlaIII/DpnII*
ITPR1	Chr3:4533821-4535043	*NlaIII/DpnII*

Supplementary Table S5. Reading, non-reading and universal primers carrying the Illumina adaptors for amplification of regions interacting with *ATXN1*, *ATXN3*, *TBP* and *ITPR1* promoter viewpoints**.** Reading and non-reading primers (in bold) carry an addition 5′ tail (red and green respectively) which anneal to the 3′ end of the forward-universal primer (in red) and index-reverse primers (in green). Three index-reverse primers with different barcodes (yellow) are used for the three replicates of each promoter viewpoint.

**Viewpoint**	**Primer**	**Sequence (5′ → 3′)**
ATXN1 promoter	Reading	TACACGACGCTCTTCCGATCT–GA–**TCAAGGATCCGACTTCATG**
	Non-reading	ACTGGAGTTCAGACGTGTGCTCTTCCGATCT-**TTTCTACTACAGTGGCGGAG**
	Forward-universal	AATGATACGGCGACCACCGAGATCTACACTCTTTCCCTACACGACGCTCTTCCGATCT
	Index-reverse	CAAGCAGAAGACGGCATACGAGATTGTTGACTGTGACTGGAGTTCAGACGTGTGCT
		CAAGCAGAAGACGGCATACGAGATACGGAACTGTGACTGGAGTTCAGACGTGTGCT
		CAAGCAGAAGACGGCATACGAGATTCTGACATGTGACTGGAGTTCAGACGTGTGCT
ATXN3 promoter	Reading	TACACGACGCTCTTCCGATCT–ACG–**TCCAGACAAATAAACATG**
	Non-reading	ACTGGAGTTCAGACGTGTGCTCTTCCGATCT-**CTCCTAGCTTCTGTTAGC**
	Forward-universal	AATGATACGGCGACCACCGAGATCTACACTCTTTCCCTACACGACGCTCTTCCGATCT
	Index-reverse	CAAGCAGAAGACGGCATACGAGATGATCTGGTGACTGGAGTTCAGACGTGTGCT
		CAAGCAGAAGACGGCATACGAGATTCAAGTGTGACTGGAGTTCAGACGTGTGCT

		CAAGCAGAAGACGGCATACGAGATCTGATCGTGACTGGAGTTCAGACGTGTGCT
TBP promoter	Reading	TACACGACGCTCTTCCGATCT-CTAGG-**ATCCGCTTCTTCTCCATG**
	Non-reading	ACTGGAGTTCAGACGTGTGCTCTTCCGATCT-**CCCGAACCGTCAGAAAAGAG**
	Forward-universal	AATGATACGGCGACCACCGAGATCTACACTCTTTCCCTACACGACGCTCTTCCGATCT
	Index-reverse	CAAGCAGAAGACGGCATACGAGATCGTGATGTGACTGGAGTTCAGACGTGTGCT
		CAAGCAGAAGACGGCATACGAGATACATCGGTGACTGGAGTTCAGACGTGTGCT
		CAAGCAGAAGACGGCATACGAGATGCCTAAGTGACTGGAGTTCAGACGTGTGCT
ITPR1 promoter	Reading	TACACGACGCTCTTCCGATCT–G–**TTTAAAGTGCAGTAACCATG**
	Non-reading	ACTGGAGTTCAGACGTGTGCTCTTCCGATCT-**CCAAACGCATTATAGCATCC**
	Forward-universal	AATGATACGGCGACCACCGAGATCTACACTCTTTCCCTACACGACGCTCTTCCGATCT
	Index-reverse	CAAGCAGAAGACGGCATACGAGATTGGTCAGTGACTGGAGTTCAGACGTGTGCT
		CAAGCAGAAGACGGCATACGAGATCACTGTGTGACTGGAGTTCAGACGTGTGCT
		CAAGCAGAAGACGGCATACGAGATATTGGCGTGACTGGAGTTCAGACGTGTGCT

Supplementary Table S6. First PCR condition for the 4C sequencing library preparation.

**Component**	***µ*l**
Buffer 1	2.5
dNTP (10 mM)	0.5
Reading primer (10 µM)	3.5
Non-reading primer (10 µM)	3.5
Expand long template polymerase	0.35
Template (150 ng)	x
MilliQ	x
**Total volume**	**25**

Supplementary Table S7. Program of the first PCR for the 4C sequencing library preparation.

**Temp (ºC)**	**Time**	**Cycles**
94	2 min	
94	15 sec	16
54	1min	
68	3 min	
68	7 min	
10	∞	

Supplementary Table S8. Second PCR condition for the 4C sequencing library preparation.

**Component**	**μl**
Buffer 1	2.5
dNTP (10 mM)	0.5
Primer mix (Forward-universal and index-reverse (5 µM))	2.5
Expand long template polymerase	0.35
First round PCR product	2.5
MilliQ	x
**Total volume**	**25**

Supplementary Table S9. Program of the second PCR for the 4C sequencing library preparation.

**Temp (ºC)**	**Time**	**Cycles**
94	2 min	
94	15 sec	20
60	1min	
68	3 min	
68	7 min	
10	∞	

Supplementary Table S10. PutE viewpoints and the 4C-template used for each viewpoint.

**PutE**	**PutE viewpoint (Hg19)**	**4C-template**
ATXN1-PutE1	chr6:16321036-16321612	*NlaIII/DpnII*
ATXN1-PutE2	chr6:16803517-16804414	*NlaIII/Csp6I*
ATXN3-PutE1	chr14:91975457-91975776	*NlaIII/DpnII*
ATXN3-PutE2	chr14:92505411-92506375	*NlaIII/Csp6I*
ATXN3-PutE3	chr14:93062668-93063126	*NlaIII/Csp6I*
ATXN3-PutE4	chr14:93215111-93216202	*NlaIII/Csp6I*
ATXN3-PutE5	chr14:93257696-93258172	*NlaIII/DpnII*
TBP-PutE1	chr6:170150992-170151453	*NlaIII/DpnII*
TBP-PutE2	chr6:170190125-170190807	*NlaIII/DpnII*
ITPR1-PutE1	chr3:3839485-3839753	*NlaIII/DpnII*
ITPR1-PutE2	chr3:4340769-4341564	*NlaIII/DpnII*
ITPR1-PutE3	chr3:4511190-4511674	*NlaIII/DpnII*
ITPR1-PutE4	chr3:4762537-4762939	*NlaIII/Csp6I*
ITPR1-PutE5	chr3:4790941-4791414	*NlaIII/DpnII*
ITPR1-PutE6	chr3:4908651-4909056	*NlaIII/DpnII*
ITPR1-PutE7	chr3:5020371-5020891	*NlaIII/DpnII*

Supplementary Table S11. Reading, non-reading and universal primers carrying the Illumina adaptors for amplification of regions interacting with the PutE viewpoints. Reading and non-reading primers (in bold) carry an addition 5′ tail (red and green, respectively) that anneals to the 3′ end of the forward-universal (in red) and index-reverse primers (in green). Two index-reverse primers with different barcodes (yellow) were used for the two biological replicates of each PutE viewpoint.

**PutE**	**Primer**	**Sequence (5′ → 3′)**
ATXN1-PutE1	Reading	TACACGACGCTCTTCCGATCT–TA–**CTCAACCAGAATCAAGGCATG**
	Non-reading	ACTGGAGTTCAGACGTGTGCTCTTCCGATCT-**AGTAGGTTAGCTGCTTGGA**
	Forward-universal	AATGATACGGCGACCACCGAGATCTACACTCTTTCCCTACACGACGCTCTTCCGATCT
	Index-reverse	CAAGCAGAAGACGGCATACGAGATGCCTAAGTGACTGGAGTTCAGACGTGTGCT
		CAAGCAGAAGACGGCATACGAGATTGGTCAGTGACTGGAGTTCAGACGTGTGCT
ATXN1-PutE2	Reading	TACACGACGCTCTTCCGATCT-A-**GGTTTTTCTAAGAAGGCATG**
	Non-reading	ACTGGAGTTCAGACGTGTGCTCTTCCGATCT-**CTTGGCTGTTACAATTCATC**
	Forward-universal	AATGATACGGCGACCACCGAGATCTACACTCTTTCCCTACACGACGCTCTTCCGATCT
	Index-reverse	CAAGCAGAAGACGGCATACGAGATAAGGCCACGTGACTGGAGTTCAGACGTGTGCT
		CAAGCAGAAGACGGCATACGAGATTCCGAAACGTGACTGGAGTTCAGACGTGTGCT
ATXN3-PutE1	Reading	TACACGACGCTCTTCCGATCT–TA-**CGTGGAGCGAGCGCCATG**
	Non-reading	ACTGGAGTTCAGACGTGTGCTCTTCCGATCT-**AGTGTGGAGAGGTCGAGGA**

	Forward-universal	AATGATACGGCGACCACCGAGATCTACACTCTTTCCCTACACGACGCTCTTCCGATCT
	Index-reverse	CAAGCAGAAGACGGCATACGAGATTGTTGACTGTGACTGGAGTTCAGACGTGTGCT
		CAAGCAGAAGACGGCATACGAGATACGGAACTGTGACTGGAGTTCAGACGTGTGCT
ATXN3-PutE2	Reading	TACACGACGCTCTTCCGATCT-ATAGG- **TTATGAAACACGGCCATG**
	Non-reading	ACTGGAGTTCAGACGTGTGCTCTTCCGATCT-**ACGTAGGAGCAGTTTCACTCT**
	Forward-universal	AATGATACGGCGACCACCGAGATCTACACTCTTTCCCTACACGACGCTCTTCCGATCT
	Index-reverse	CAAGCAGAAGACGGCATACGAGATCGTGATGTGACTGGAGTTCAGACGTGTGCT
		CAAGCAGAAGACGGCATACGAGATACATCGGTGACTGGAGTTCAGACGTGTGCT
ATXN3-PutE3	Reading	TACACGACGCTCTTCCGATCT-TGG-**TCTATGAATACCCACATG**
	Non-reading	ACTGGAGTTCAGACGTGTGCTCTTCCGATCT-**TTGGAATTACCCTTGGAGTC**
	Forward-universal	AATGATACGGCGACCACCGAGATCTACACTCTTTCCCTACACGACGCTCTTCCGATCT
	Index-reverse	CAAGCAGAAGACGGCATACGAGATCGGGACGGGTGACTGGAGTTCAGACGTGTGCT
		CAAGCAGAAGACGGCATACGAGATGTGCGGACGTGACTGGAGTTCAGACGTGTGCT
ATXN3-PutE4	Reading	TACACGACGCTCTTCCGATCT-ATCA-**ATCCATTCGAGTGCCATG**
	Non-reading	ACTGGAGTTCAGACGTGTGCTCTTCCGATCT-**ACAAGGTTGGCACACAGGT**

	Forward-universal	AATGATACGGCGACCACCGAGATCTACACTCTTTCCCTACACGACGCTCTTCCGATCT
	Index-reverse	CAAGCAGAAGACGGCATACGAGATATCCACTCGTGACTGGAGTTCAGACGTGTGCT
		CAAGCAGAAGACGGCATACGAGATATATCAGTGTGACTGGAGTTCAGACGTGTGCT
ATXN3-PutE5	Reading	TACACGACGCTCTTCCGATCT-GA-**CTGTGACACGTTACCATG**
	Non-reading	ACTGGAGTTCAGACGTGTGCTCTTCCGATCT-**AAACTTCATCCTTAAGCTCT**
	Forward-universal	AATGATACGGCGACCACCGAGATCTACACTCTTTCCCTACACGACGCTCTTCCGATCT
	Index-reverse	CAAGCAGAAGACGGCATACGAGATCGGGACGGGTGACTGGAGTTCAGACGTGTGCT
		CAAGCAGAAGACGGCATACGAGATGTGCGGACGTGACTGGAGTTCAGACGTGTGCT
TBP-PutE1	Reading	TACACGACGCTCTTCCGATCT–TA-**TGCCTCCTTCTCGAACATG**
	Non-reading	ACTGGAGTTCAGACGTGTGCTCTTCCGATCT-**AGTCGATTGGAAGGGTGGTT**
	Forward universal	AATGATACGGCGACCACCGAGATCTACACTCTTTCCCTACACGACGCTCTTCCGATCT
	Index-reverse	CAAGCAGAAGACGGCATACGAGATGATCTGGTGACTGGAGTTCAGACGTGTGCT
		CAAGCAGAAGACGGCATACGAGATTCAAGTGTGACTGGAGTTCAGACGTGTGCT
TBP-PutE2	Reading	TACACGACGCTCTTCCGATCT–GCAC-**GTAGGAGTTCCGACGCATG**

	Non-reading	ACTGGAGTTCAGACGTGTGCTCTTCCGATCT-**CCTTTGCGAATCGATGGGGA**
	Forward-universal	AATGATACGGCGACCACCGAGATCTACACTCTTTCCCTACACGACGCTCTTCCGATCT
	Index-reverse	CAAGCAGAAGACGGCATACGAGATAAGCTAGTGACTGGAGTTCAGACGTGTGCT
		CAAGCAGAAGACGGCATACGAGATGTAGCCGTGACTGGAGTTCAGACGTGTGCT
ITPR1-PutE1	Reading	TACACGACGCTCTTCCGATCT–CG–**TATCAGACAAACTCCATG**
	Non-reading	ACTGGAGTTCAGACGTGTGCTCTTCCGATCT-**CAGTCGGAGCAATTAATGAT**
	Forward-universal	AATGATACGGCGACCACCGAGATCTACACTCTTTCCCTACACGACGCTCTTCCGATCT
	Index-reverse	CAAGCAGAAGACGGCATACGAGATCACTGTGTGACTGGAGTTCAGACGTGTGCT
		CAAGCAGAAGACGGCATACGAGATATTGGCGTGACTGGAGTTCAGACGTGTGCT
ITPR1-PutE2	Reading	TACACGACGCTCTTCCGATCT-C-**TTAGCTATTATTTCCCATG**
	Non-reading	ACTGGAGTTCAGACGTGTGCTCTTCCGATCT-**GGTTTCACCGAATTGACC**
	Forward-universal	AATGATACGGCGACCACCGAGATCTACACTCTTTCCCTACACGACGCTCTTCCGATCT
	Index-reverse	CAAGCAGAAGACGGCATACGAGATAAGGCCACGTGACTGGAGTTCAGACGTGTGCT
		CAAGCAGAAGACGGCATACGAGATTCCGAAACGTGACTGGAGTTCAGACGTGTGCT
ITPR1-PutE3	Reading	TACACGACGCTCTTCCGATCT-G-**CCTAGGTCATACTTCATG**

	Non-reading	ACTGGAGTTCAGACGTGTGCTCTTCCGATCT-**GATGGAACTTTGTTGGTTCT**
	Forward-universal	AATGATACGGCGACCACCGAGATCTACACTCTTTCCCTACACGACGCTCTTCCGATCT
	Index-reverse	CAAGCAGAAGACGGCATACGAGATTACGTACGGTGACTGGAGTTCAGACGTGTGCT
		CAAGCAGAAGACGGCATACGAGATAAGCTAGTGACTGGAGTTCAGACGTGTGCT
ITPR1-PutE4	Reading	TACACGACGCTCTTCCGATCT-A-**AAGTTGTATAATTTGGTCATG**
	Non-reading	ACTGGAGTTCAGACGTGTGCTCTTCCGATCT-**GCAGTGAGCCTAAATTCCA**
	Forward-universal	AATGATACGGCGACCACCGAGATCTACACTCTTTCCCTACACGACGCTCTTCCGATCT
	Index-reverse	CAAGCAGAAGACGGCATACGAGATGTAGCCGTGACTGGAGTTCAGACGTGTGCT
		CAAGCAGAAGACGGCATACGAGATTACAAGGTGACTGGAGTTCAGACGTGTGCT
ITPR1-PutE5	Reading	TACACGACGCTCTTCCGATCT–GC–**GCAACTGAAAAGGTGCCATG**
	Non-reading	ACTGGAGTTCAGACGTGTGCTCTTCCGATCT-**GGGCCTCCATATACACTGA**
	Forward-universal	AATGATACGGCGACCACCGAGATCTACACTCTTTCCCTACACGACGCTCTTCCGATCT
	Index-reverse	CAAGCAGAAGACGGCATACGAGATGATCTGGTGACTGGAGTTCAGACGTGTGCT
		CAAGCAGAAGACGGCATACGAGATTCAAGTGTGACTGGAGTTCAGACGTGTGCT

ITPR1-PutE6	Reading	TACACGACGCTCTTCCGATCT—**TTTTTGTGAGCCTTTCATG**
	Non-reading	ACTGGAGTTCAGACGTGTGCTCTTCCGATCT-**CAAACGGATTTTCTCTGAACTC**
	Forward-universal	AATGATACGGCGACCACCGAGATCTACACTCTTTCCCTACACGACGCTCTTCCGATCT
	Index-reverse	CAAGCAGAAGACGGCATACGAGATCTGATCGTGACTGGAGTTCAGACGTGTGCT
		CAAGCAGAAGACGGCATACGAGATTGTTGACTGTGACTGGAGTTCAGACGTGTGCT
ITPR1-PutE7	Reading	TACACGACGCTCTTCCGATCT—**CAACACGTGAGACTCATG**
	Non-reading	ACTGGAGTTCAGACGTGTGCTCTTCCGATCT-**AGTTCCCGTCTCCTGGGA**
	Forward-universal	AATGATACGGCGACCACCGAGATCTACACTCTTTCCCTACACGACGCTCTTCCGATCT
	Index-reverse	CAAGCAGAAGACGGCATACGAGATACGGAACTGTGACTGGAGTTCAGACGTGTGCT
		CAAGCAGAAGACGGCATACGAGATTCTGACATGTGACTGGAGTTCAGACGTGTGCT

Supplementary Table S12. Primers used to amplify PutEs of *ITPR1* for cloning into the pGL4.23 Firefly luciferase reporter vector. Primer sequences are shown in bold and restriction sites in green.

**PutEs**	**Cloned region (Hg19)**	**Primers Sequence (5′ → 3′)**		
ITPR1-PutE1	chr3:3840264-3842199	A	Forward	AAAAGATATC**GCTTTCGCTTAAAGCCGATTCCC**
			Reverse	TTTTAAGCTT**AGATCCTTCCCAAGGCTCTGACTTC**
	chr3:3842041-3843945	B	Forward	AAAAGATATC**AGCTGGAAAGAGGGCCAAGAGGAG**
			Reverse	TTTTAAGCTT**GTCGCACAACGCCAAGAGGTGAAC**
ITPR1-PutE4	chr3:4762029-4764147	Forward		AAAACTCGAG**GGCTTAGGAGATCAGGTGATGAC**
		Reverse		TTTTGATATC**GGTGAAGCCCTCATTGAACATGTG**
ITPR1-PutE5	chr3:4790170-4792499	A	Forward	AAAACTCGAG**TGCTGGTGTCTTTCTTGTGGGTAG**
			Reverse	TTTTGATATC**TTTCCGGAGACAGCTCCAAGAATC**
	chr3:4792321-4794208	B	Forward	AAAACTCGAG**GGTGGACTGCGTTGTTGCTATGG**
			Reverse	TTTTGATATC**GAATATGCAGCACACCGCAGACC**
ITPR1-PutE6	chr3:4908637-4910722	Forward		AAAACTCGAG**ATACTGCGTGCGGTCATGAAAGGC**
		Reverse		TTTTGATATC**CCTGACCACAGCAGTAGCTTCATAG**

Supplementary Table S13. Primers used to amplify the region overlapping between the CNV deletion and the *ITPR1* 4C-contact peak for cloning into the pGL4.23 Firefly luciferase reporter vector. Primer sequences are shown in bold and restriction sites in green.

**Region of interest**	**Cloned region (Hg19)**	**Primers Sequence (5′ → 3′)**	
ITPR1-contact peak-deletion	chr3:3837002-3838389	Forward	AAAACTCGAG**GTCTATGAGCTGGTCACATCATCC**
		Reverse	TTTTGATATC**CGATTGGCTGTGTTGAGACACTAC**

Supplementary Table S14. Primers used for Sanger sequencing to confirm the break point of the CNV identified in the patient.

**Primers**	**Sequence Sequence (5′ → 3′)**
Forward-1	GAGTAGGGAAGAAACGCACCCAATC
Forward-2	CAAGCTCACTCTGACTGGGTATG
Forward-3	CTGCCCTACCCAATATGGTTGTC
Forward-4	GTGAGCTTGTAAACGGTCCAAGG
Forward-5	TTGCCGACCGGAGAGTCTGAAATC
Forward-6	CACGTGGCCAACTCCCTAACTTC
Universal-reverse	TAGGAGGCCAGCTAGGCACAATG

Supplementary Table S15. qPCR primers used to measure the expression of *ITPR1* and *GAPDH* and *Beta-actin* in whole blood.

**Gene**	**Primers Sequence (5′ → 3′)**	
ITPR1-A	Forward	GTTTCAGCCCTCAGTGGACC
	Reverse	CCTTCAGGCACAGAGACCAG
ITPR1-B	Forward	CCAGTTCAAAAGCCCTGTG
	Reverse	TGTTCCAATACCCTGCTC
GAPDH	Forward	ATGGGGAAGGTGAAGGTCG
	Reverse	GGGGTCATTGATGGCAACAATA
Beta-actin	Forward	AGCCTCGCCTTTGCCGA
	Reverse	GCGCGGCGATATCATCATC

Supplementary Table S16. List of genes mentioned in this study, along with their chromosomal positions and known functions (https://www.genecards.org/(accessed on 2 October 2024)).

Gene	Chromosomal position (Hg19)	Known function
ATXN1	chr6:16,299,343-16,761,721	Involved in transcription regulation by binding to DNA and RNA
ATXN3	chr14:92,524,896-92,572,926	Deubiquitinating enzyme
TBP	chr6:170,863,457-170,881,947	General transcription factor
ITPR1	chr3:4,535,032-4,889,190	Calcium channel
PPP4R3A	chr14:91,923,852-91,976,898	Regulating the cellular processes, including DNA damage repair
TRIP11	chr14:92,432,335-92,506,403	Maintenance of Golgi structure and function
DYNLT2	chr6:170,140,205-170,151,668	Enables dynein intermediate chain binding activity
LINC00242	chr6:170,188,886-170,198,921	Survival related eRNA
LINC00574	chr6:170,190,169-170,202,969	Chemoresistance-associated long non-coding RNA
SUMF1	chr3:4,402,830-4,508,953	Catalyzes the hydrolysis of sulfate esters
SETMAR	chr3:4,344,988-4,358,949	Regulates gene expression
BHLHE40-AS1	chr3:4,938,493-5,021,646	Noncoding RNA acting as a transcription factor

Supplementary figures

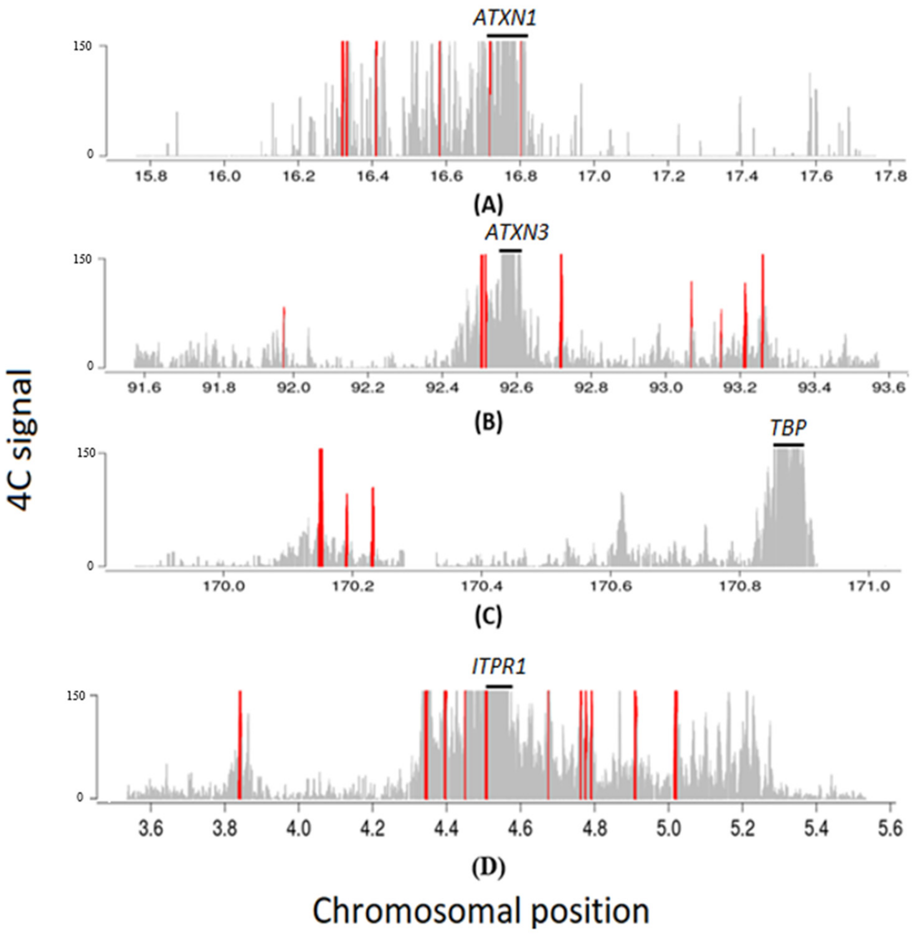

Supplementary Figure S1. 4C-seq data of *ATXN1*, *ATXN3*, *TBP* and *ITPR1* promoter showing genomic regions significantly interacting with the promoters (4C-contact peaks). The location of the promoter viewpoints is shown as a black line and the 4C-contact peaks as red bars. (**A**) *ATXN1* promoter has six significant 4C-contact peaks. (**B**) *ATXN3* promoter has eight significant 4C-contact peaks. (**C**) *TBP* promoter has three significant 4C-contact peaks. (**D**) *ITPR1* promoter has eleven significant 4C-contact peaks.

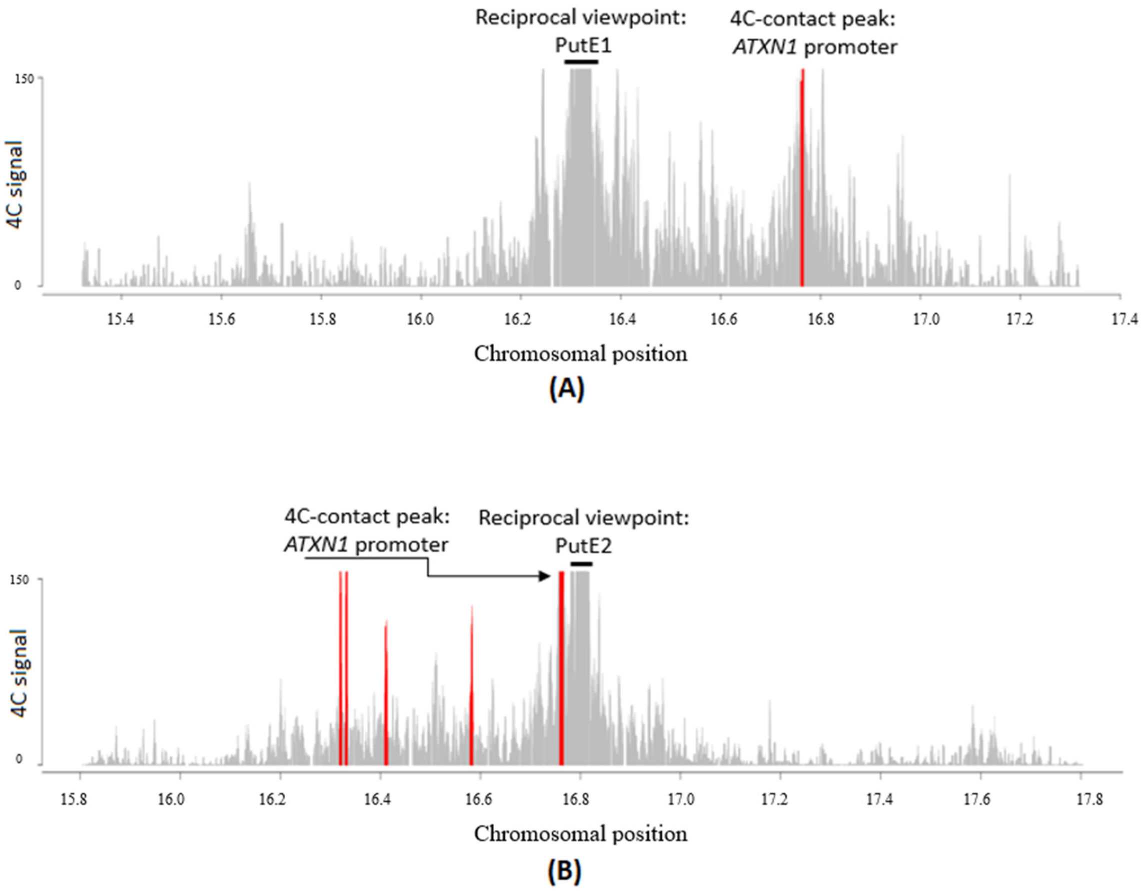

Supplementary Figure S2. Reciprocal 4C-seq for the PutEs of *ATXN1*. (**A**) PutE1 is selected as a reciprocal viewpoint (chr6:16319229-16323196) and *ATXN1* promoter (chr6:16759520-16767014) is a 4C-contact peak for PutE1. (**B**) PutE2 is selected as a reciprocal viewpoint (chr6:16803696-16804514) and *ATXN1* promoter (chr6:16758209-16766894) is a 4C-contact peak for PutE2. Based on the Eukaryotic Promoter Database, the location of the *ATXN1* promoter is chr6:16759685–1676368. Black lines show the location of the reciprocal viewpoint. Red bars indicate the significant 4C-contact peaks.

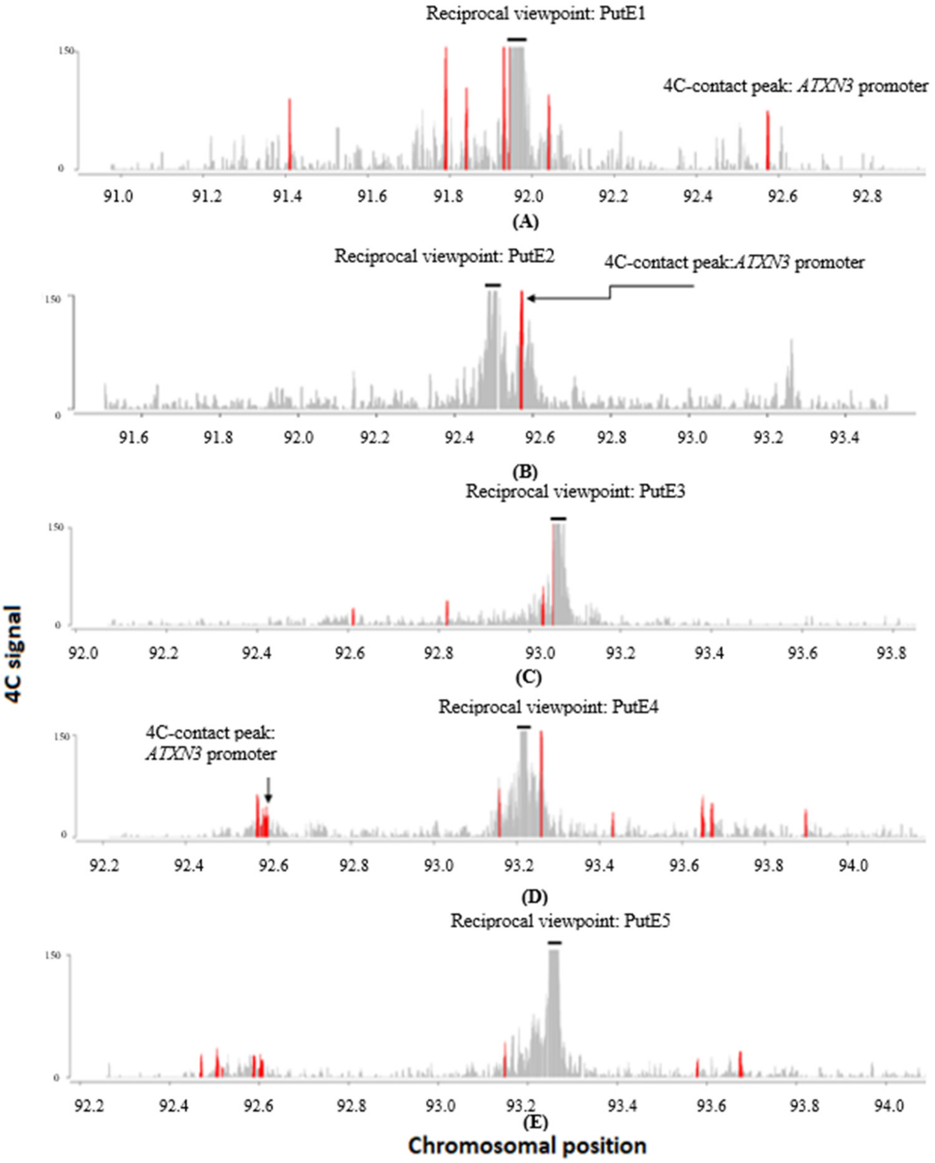

Supplementary Figure S3. Reciprocal 4C-seq for PutEs of *ATXN3*. (**A**) PutE1 is selected as a reciprocal viewpoint (chr14:91975209-91975777), and *ATXN3* promoter (chr14:92570837-92577020) is a 4C-contact peak for PutE1. (**B**) PutE2 is selected as a reciprocal viewpoint (chr14:92505300-92508216), and *ATXN3* promoter (chr14:92567911-92574975) is a 4C-contact peak for PutE2. (**C**) PutE3 is selected as a reciprocal viewpoint (chr14:93069393-93070523), but *ATXN3* promoter is not a 4C-contact peak for PutE3. (**D**) PutE4 is selected as a reciprocal viewpoint (chr14:93214153-93215641), and *ATXN3* promoter (chr14:92570462-92599948) is a 4C-contact peak for PutE4. (**E**) PutE5 is selected as a reciprocal viewpoint (chr14: chr14:93259723-93261534), but *ATXN3* promoter is not a 4C-contact peak for PutE5. Based on the Eukaryotic Promoter Database, the location of the *ATXN3* promoter is chr14:92570908–92574908. Black lines show the location of the reciprocal viewpoint. Red bars indicate the significant 4C-contact peaks.

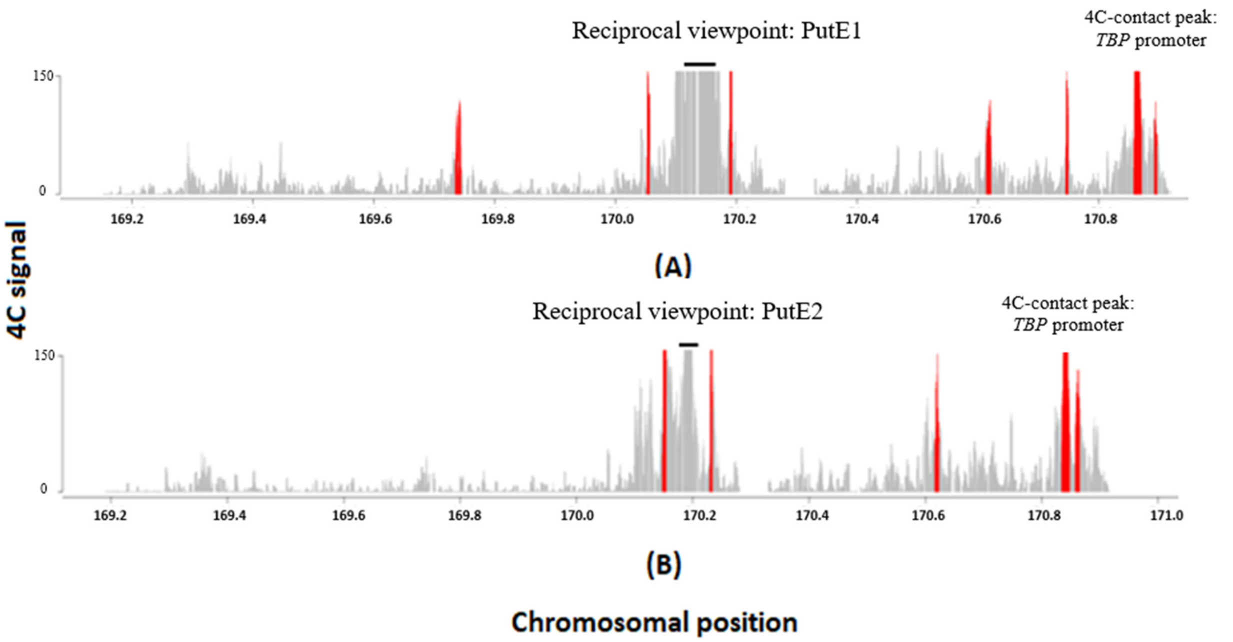

Supplementary Figure S4. Reciprocal 4C-seq for PutEs of *TBP*. (**A**) PutE1 is selected as a reciprocal viewpoint (chr6:170151018-170153022), and *TBP* promoter (chr6:170857754-170871249) is a 4C-contact peak for PutE1. (**B**) PutE2 is selected as a reciprocal viewpoint (chr6:170189867-170191217), and *TBP* promoter (chr6:170857849-170865809) is a 4C-contact peak for PutE2. Based on the Eukaryotic Promoter Database, the location of the *TBP* promoter is chr6:170861456–170865456. Black lines show the location of the reciprocal viewpoint. Red bars indicate the significant 4C-contact peaks.

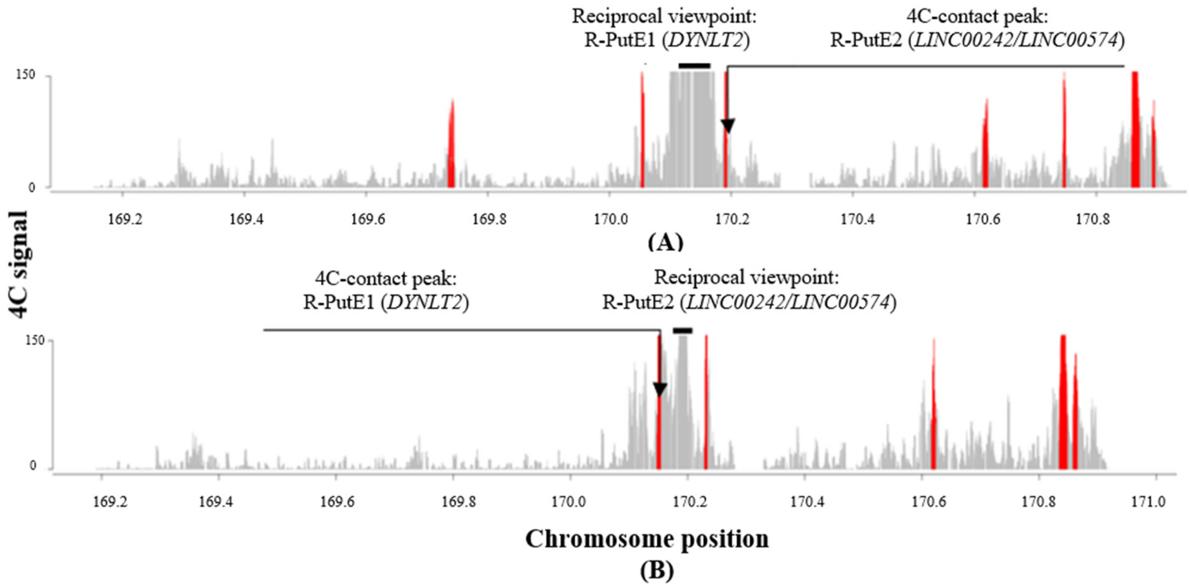

Supplementary Figure S5. Reciprocal 4C-seq showing an interaction between R-PutE1 and R-PutE2 of *TBP*. (**A**) R-PutE1, which encompasses *DYNLT2*, is selected as a reciprocal viewpoint (chr6:170151018-170153022) and R-PutE2, which encompasses *LINC00242/LINC00574* (chr6:170188785-170193422) is a 4C-contact peak for R-PutE1. (**B**) R-PutE2 which encompasses *LINC00242/LINC00574* is selected as a reciprocal viewpoint (chr6:170189867-170191217), and R-PutE1 which encompasses *DYNLT2* (chr6:170147727-170154438) is a 4C-contact peak for R-PutE2. Black lines show the location of the reciprocal viewpoint. Red bars indicate the significant 4C-contact peaks.

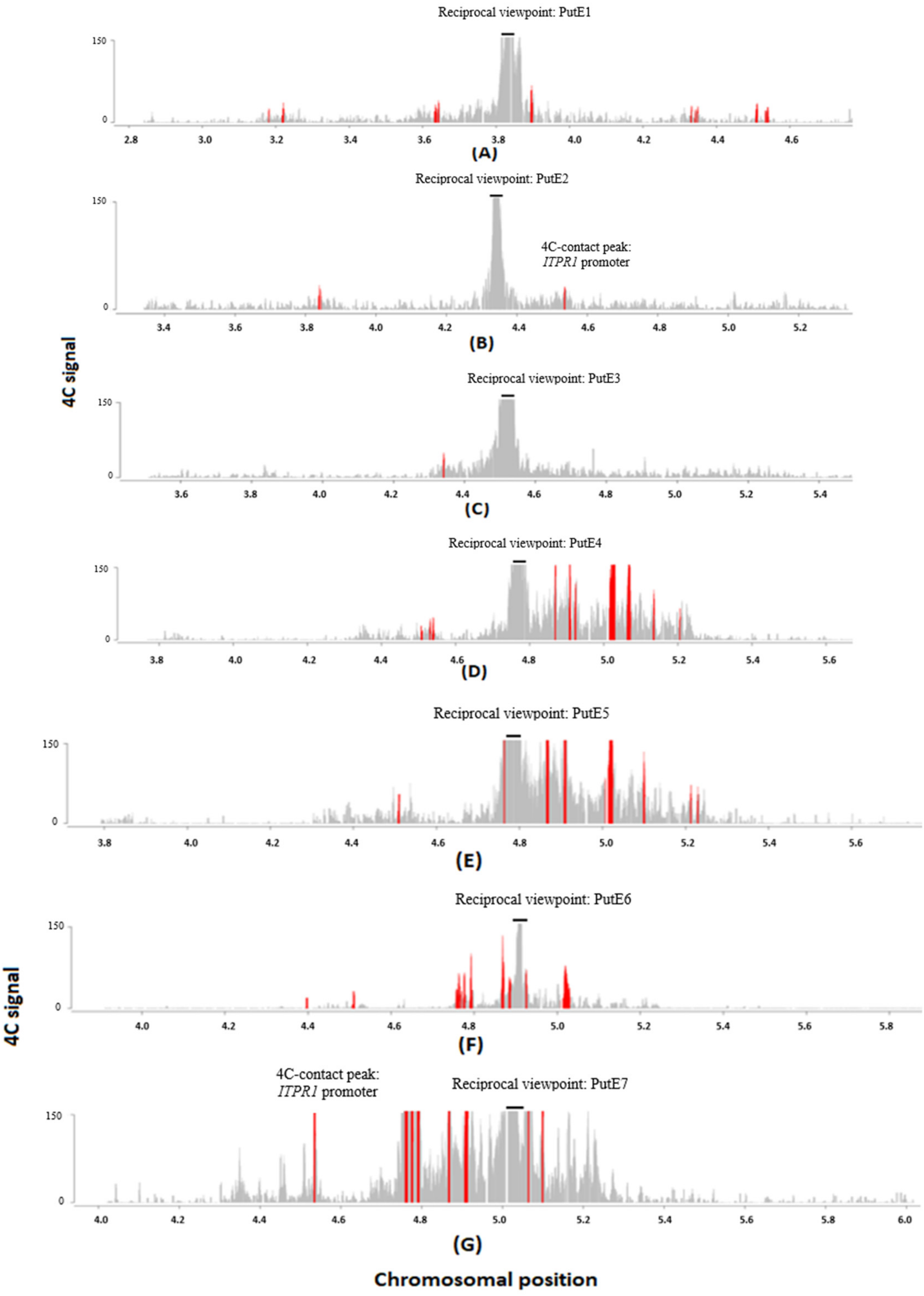

Supplementary Figure S6. Reciprocal 4C-seq for the PutEs of *ITPR1*. (**A**) PutE1 is selected as a reciprocal viewpoint (chr3:3839946-3843237), but *ITPR1* promoter is not a 4C-contact peak for PutE1. (**B**) PutE2 is selected as a reciprocal viewpoint (chr3:4344258-4346242), and *ITPR1* promoter (chr3:4533825-4538002) is a 4C-contact peak for PutE2. (**C**) PutE3 is selected as a reciprocal viewpoint (chr3:4508178-4509659), but *ITPR1* promoter is not a 4C-contact peak for PutE3. (**D**) PutE4 is selected as a reciprocal viewpoint (chr3:4761716-4763874), but *ITPR1* promoter is not a 4C-contact peak for PutE4. (**E**) PutE5 is selected as a reciprocal viewpoint (chr3:4791018-4794112), but *ITPR1* promoter is not a 4C-contact peak for PutE5.

(**F**) PutE6 is selected as a reciprocal viewpoint (chr3:4908570-4911348), but *ITPR1* promoter is not a 4C-contact peak for PutE6. (**G**) PutE7 is selected as a reciprocal viewpoint (chr3:5018875-5022761) and *ITPR1* promoter (chr3:4533360-4537206) is a 4C-contact peak for PutE7. Based on the Eukaryotic Promoter Database, the location of the *ITPR1* promoter is chr3:4533031–4537031. Black lines show the location of the reciprocal viewpoint. Red bars indicate the significant 4C-contact peaks.

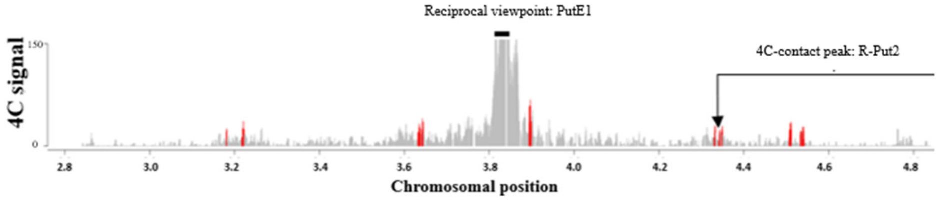
Reciprocal viewpoint: PutE1
4C-contact peak: R-Put2
4C signal
150
0
2.8
3.0
3.2
3.4
3.6
3.8
4.0
4.2
4.4
4.6
4.8
Chromosomal position

(A)

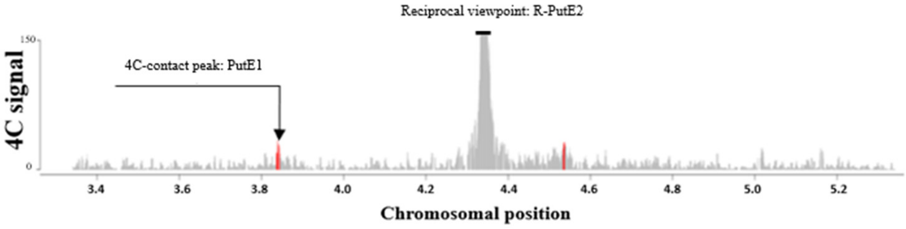
Reciprocal viewpoint: R-PutE2
4C-contact peak: PutE1
4C signal
150
0
3.4
3.6
3.8
4.0
4.2
4.4
4.6
4.8
5.0
5.2
Chromosomal position

(B)

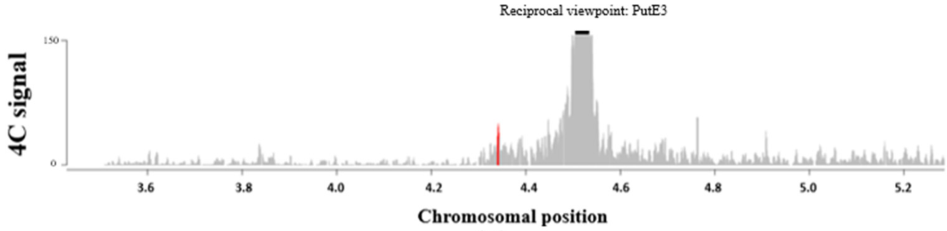
Reciprocal viewpoint: PutE3
4C signal
150
0
3.6
3.8
4.0
4.2
4.4
4.6
4.8
5.0
5.2
Chromosomal position

(C)

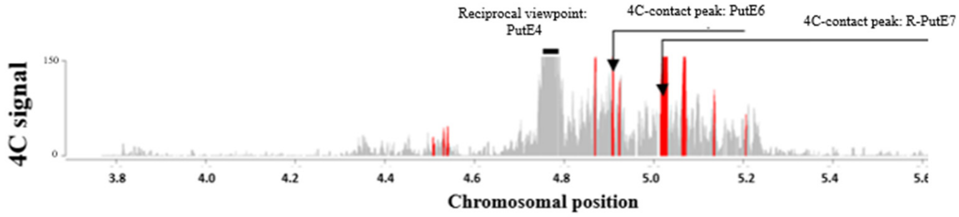
Reciprocal viewpoint:
PutE4
4C-contact peak: PutE6
4C-contact peak: R-PutE7
4C signal
150
0
3.8
4.0
4.2
4.4
4.6
4.8
5.0
5.2
5.4
Chromosomal position

(D)

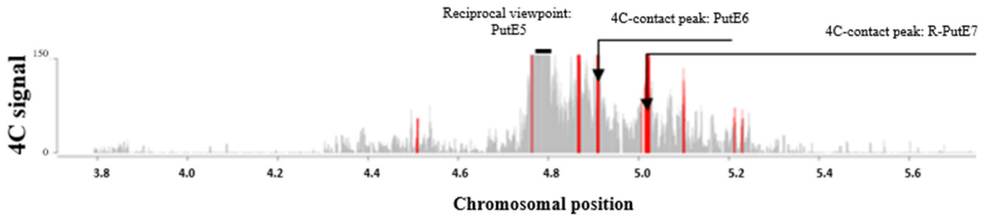

(E)

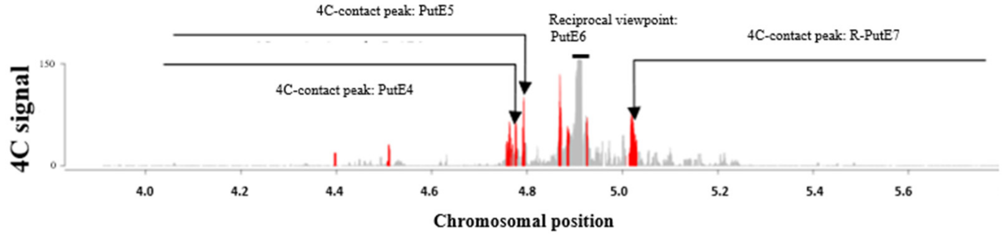

(F)

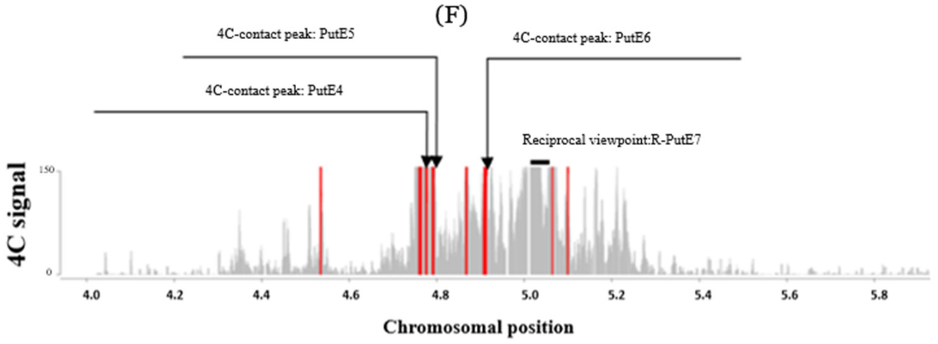

(G)

Supplementary Figure S7. Reciprocal 4C-seq for the PutEs of *ITPR1* showing interactions between the PutEs/R-PutEs. (**A**) PutE1 interacts with R-PutE2. (**B**) R-PutE2 interacts with PutE1. (**C**) PutE3 does not interact with other PutEs/R-PutEs. (**D**) PutE4 interacts with PutE6 and R-PutE7. (**E**) PutE5 interacts with PutE6 and R-PutE7. (**F**) PutE6 interacts with PutE4, PutE5 and R-PutE7. (**G**) R-PutE7 interacts with PutE4, PutE5 and PutE6.

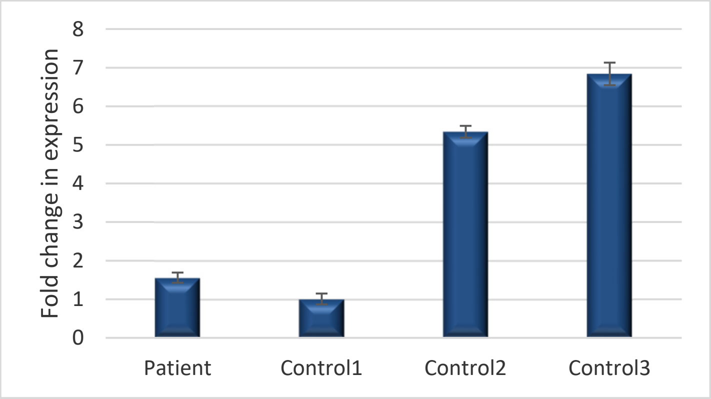

Supplementary Figure S8. Expression analysis of *ITPR1* in blood from the SCA patient and three healthy individuals using a second set of primers to measure *ITPR1* expression levels for confirmation. The delta-delta Ct method is used to determine the relative fold change in expression levels. Expression value for control 1 is set to 1, with other individuals compared to control 1. The relative fold change in expression for each individual is based on the means of three independent replicates. Standard deviations are shown as error bars.
